# Velocity Loss as an Indicator of Resistance Training Volume in Women

**DOI:** 10.5114/jhk/190387

**Published:** 2024-12-06

**Authors:** Beatriz Bachero-Mena, Luis Rodiles-Guerrero, Juan Sánchez-Valdepeñas, Pedro J. Cornejo-Daza, Clara Cano-Castillo, Fernando Pareja-Blanco, Miguel Sánchez-Moreno

**Affiliations:** 1 Department of Human Movement and Sport Performance, University of Seville, Seville, Spain.; 2 Science Based Training Research Group, Physical Performance & Sports Research Center (CIRFD), Universidad Pablo de Olavide, Seville, Spain.; 3 Physical Education and Sports Department, Cardenal Spinola CEU Andalucia University, Bormujos, Seville, Spain.; 4 Department of Physical Education, University of Seville, Seville, Spain.

**Keywords:** female athletes, velocity-based training, bench-press, level of effort, task failure

## Abstract

This study aimed to analyze the evolution of repetition velocity throughout a set until failure in the bench-press exercise and to analyze the relationships between the percentage of performed repetitions (%Rep) regarding the maximum number of repetitions that can be completed (MNR) and the percentage of velocity loss (VL) in women. Sixteen women performed one set until failure with four different intensities (50%, 60%, 70%, and 80% of one-repetition maximum, 1RM). Two-testing sessions were performed with 50% and 80% 1RM to evaluate data stability. The level of significance was set at p ≤ 0.05. A close relationship was observed between the magnitude of VL and the %Rep (R^2^ = 0.85–0.92) and a low standard error of the estimation (6.85–9.81%). Regarding reliability, the MNR showed a coefficient of variation (CV) of 16.1% and 20.8% for 50% and 80% 1RM, respectively. Regarding the %Rep for a given VL (from 15% VL), CVs were: 6.3–19.6%, being higher when VL reached in the set was lower. This study shows the usefulness of monitoring VL to estimate, with considerable precision, the %Rep in women. However, the %Rep when a given VL was reached revealed only satisfactory absolute reliability from a certain VL threshold (>15% VL).

## Introduction

Resistance training (RT) is becoming more popular among women as a result of the significant growing number of women participating in sports training activities as well as the increase in women’s sporting events. Long-term systematic RT has been proven to increase skeletal muscle size and strength across a spectrum of age groups, encompassing both male and female subjects (Ahtiainen et al., 2016). There is evidence that RT can have many benefits for women, in terms of improved muscle, cardiovascular performance, and in terms of increasing bone density (Gentil et al., 2016; Kelly et al., 2008; Xu et al., 2016). Moreover, RT is beneficial for women regarding body composition, maternal health during pregnancy, and quality of life in elderly and breast cancer patients (Barbalho et al., 2017; Dos Santos et al., 2017; Paoli et al., 2015; Perales et al., 2016). Additionally, it has been demonstrated that RT can treat and lessen the risk of several chronic diseases (Hurley et al., 2011).

Sex differences in muscle size and distribution have been documented (Janssen et al., 2000). The proportion of total and lean body mass is lower in females, and they are more likely to have greater percentages of body fat and shorter muscle fiber diameters than males (Roberts et al., 2018). Also, research has observed a higher prevalence of type I fibers in the vastus lateralis and biceps brachii muscles in women compared to men (Miller et al., 1993; Roberts et al., 2018). These differences in the physiological characteristics may contribute to elucidating disparities observed in strength or muscle growth between men and women. Hence, this could have an impact on the design of training programs and the subsequent adaptations. Previous research has indicated that females can perform similar RT programs to males and achieve similar outcomes after an RT intervention (Gentil et al., 2016). Notwithstanding, it has been observed that their response to a single training session differs, particularly in terms of fatigability and muscle recovery (Ansdell et al., 2019; Chukhlantseva et al., 2023; Häkkinen, 1994; Hill et al., 2018; Walker et al., 2022).

The velocity-based training approach is an RT method that facilitates the regulation of training intensity (González-Badillo and Sánchez-Medina, 2010). In addition, monitoring velocity loss (VL) throughout a set provides accurate information about mechanical, metabolic, neuromuscular, and hormonal responses and perceived soreness (Pareja-Blanco et al., 2017; Sánchez-Medina et al., 2011; Tsoukos and Bogdanis, 2023; Weakley et al., 2024). In this regard, close relationships (R^2^ = 0.92–0.97) between VL and the percentage of performed repetitions (%Rep) have been observed in several resistance exercises: a bench-press (BP), a full-squat, a shoulder press, and a prone bench pull performed on a Smith machine in male populations (González-Badillo et al., 2017; Rodríguez-Rosell et al., 2020; Sánchez-Moreno et al., 2021; Hernández-Belmonte et al., 2022). Likewise, an acceptable inter-individual variability and high reliability in the %Rep for a given VL were reported (González-Badillo et al., 2017; Hernández-Belmonte et al., 2022). Consequently, the %Rep completed for a particular VL remains consistent across male individuals, irrespective of the maximal number of repetitions (MNR) achievable or the strength levels. However, the validity and reliability of these relationships have been questioned when referring to the free-weight back squat exercise (Jukic et al., 2023). On the other hand, several recent studies have examined the accuracy of objective and subjective estimations in determining the level of effort or the actual number of repetitions in reserve (Morán-Navarro et al., 2019; Hickmott et al., 2024). In this regard, Halperin et al. (2022), in their recent review, observed an imperfection in the ability to predict proximity to task failure by participants independently of their training background and suggested that prediction accuracy can be improved if it is provided closer to task failure, when using heavier loads, or in later sets.

Practitioners can benefit from these findings to accurately estimate the %Rep already accomplished and, consequently, determine the number of remaining repetitions available upon reaching defined VL magnitude within an exercise set. However, according to our knowledge, very few studies have employed velocity-based training to prescribe RT programs in women (Rebelo et al., 2023), and there has been no previous study analyzing the relationship between VL and the %Rep in the female population. Due to the differences between sexes regarding fatigability and muscle recovery (Ansdell et al., 2019; Häkkinen, 1994; Hill et al., 2018; Linnamo et al., 1998), it is unknown whether women would show a distinct behavior in the VL-%Rep relationship. For this reason, this study analyzed the evolution of repetition velocity in the BP exercise throughout a set performed until failure, and the relationships between the %Rep and the percentage of VL with four different intensities (50%, 60%, 70% and 80% 1RM) in women in order to enhance our understanding of female responses and refine exercise training protocols accordingly.

## Methods

### Participants

Sixteen young women (age 22.2 ± 2.3 years; body height 1.65 ± 0.04 m; body mass 62.2 ± 6.0 kg) with RT experience in the BP exercise (at least one year; 1RM for the BP: 42.7 ± 8.1 kg, and 0.69 ± 0.15 kg•kg of body mass^−1^) participated in the study. Upon enrollment, participants were asked queries regarding their menstrual cycle to guarantee precise data collection and documentation for potential future requirements (14 participants were naturally menstruating, two were using hormonal contraceptives, and none of them were amenorrheic). Participants were informed that if they experienced any discomfort during the study period that could affect performance, testing sessions would be rescheduled as needed. Participants were healthy and received comprehensive information regarding the study's procedures, potential risks, and benefits. Prior to the tests, all participants provided written informed consent. Additionally, participants confirmed that they were not using any drugs, medications or dietary supplements that could impact physical performance. The study was approved by the Institutional Review Board of Hospitales Universitarios Virgen Macarena-Virgen del Rocío (protocol code 1547-N-19; approval date: 11 October 2019), and conducted in accordance with the Declaration of Helsinki.

### Study Design

A cross-sectional design was undertaken to investigate the change in velocity during a single set performed until task failure, defined as the point where the activated muscles were incapable of completing another repetition in an appropriate range of motion (Jenkins et al., 2015), with four intensities (50, 60, 70, and 80% 1RM) in the BP exercise. One testing session was carried out for each intensity and an additional testing session was carried out for 50% and 80% 1RM to evaluate data stability, making a total of six testing sessions (two with 50%, one with 60%, one with 70%, and two with 80% 1RM). Initial familiarization sessions were carried out two weeks before the first trial. Those sessions consisted of performing the BP exercise with different loads and repetitions, always at maximal intended velocity. Following familiarization, an incremental loading test session was carried out to establish the 1RM and describe the strength characteristics of the study sample. The sessions were randomly performed and separated by 5–7 days. Participants were instructed to refrain from engaging in vigorous physical activity for a minimum of two days prior to the tests. All sessions were conducted in a research facility under controlled environmental conditions (20ºC and 60% humidity) and direct supervision of the investigators.

### Testing Procedures

#### Incremental Loading Test

The BP was performed with participants reclined on a bench on a Smith machine (Bench Fitness Line and Multipower Fitness Line, Peroga, Murcia, Spain), with their feet positioned on the floor. Participants freely selected their preferred grip width. The selected bar width grip was noted and replicated in subsequent sessions. The eccentric phase of the movement was executed with control, followed by a one-second pause with the bar resting on the chest. This approach aimed to mitigate the influence of the rebound effect and facilitate more consistent measurements (Pallares et al., 2014). Subsequently, the concentric phase was executed at maximum intended velocity as soon as the command was received. A linear velocity transducer was employed to record the mean propulsive velocity (MPV) of each repetition (T-Force System, Ergotech, Murcia, Spain). MPV is the mean velocity achieved during the concentric phase, excluding the deceleration phase where measured acceleration surpasses gravity (−9.81 m•s^−2^) (Sánchez-Medina et al., 2010). The initial load consisted of one set of 3 repetitions with 10 kg and the load was progressively increased in 5-kg increments until the attained MPV was ≤0.30 m•s^−1^ (load ≥ 90% 1RM). Once this velocity was attained, the test was concluded. The value of the 1RM was estimated through the general load-velocity relationship for the BP previously reported in women (Pareja-Blanco et al., 2020). Three repetitions were executed for light (>0.80 m•s^−1^), two for medium (0.80−0.60 m•s^−1^), and only one for heavy (<0.60 m•s^−1^) loads. Inter-set recovery periods were of 3 minutes. The warm-up consisted of 5 min of easy-paced jogging, 2 min of joint mobilization exercises, and two sets of 6 repetitions with 10 kg. Only the fastest repetition with each load was considered for subsequent analysis.

#### Repetitions to Failure Sessions

During each session, participants completed one set in the BP exercise to failure at the prescribed intensity. Participants were required to complete as many repetitions as possible until muscle failure, performing each repetition at maximum concentric velocity. The exercise execution technique and the instruments used for data collection were the same as described in the incremental loading test. Relative loads were obtained through the general load-velocity relationship for the BP previously reported in women (Pareja-Blanco et al., 2020). Hence, MPV achieved during the fastest repetition of the set (typically the first) was utilized as an approximation of %1RM. The references used were: 0.79 m•s^−1^, 0.67 m•s^−1^, 0.55 m•s^−1^, and 0.43 m•s^−1^, for 50, 60, 70 and 80% 1RM, respectively. The load (in kilograms) was selected to match the velocity targeted for the intended %1RM. A 0.03m•s^−1^ range was utilized since it has been shown (Courel-Ibáñez et al., 2019) that this is the smallest detectable change in MPV when using the setting of this study (i.e., BP exercise, Smith Machine, and T-Force System). The general warm-up described in the incremental loading test section was implemented before each session. In addition, a specific warm-up for each intensity protocol involving the BP exercise was conducted, comprising: 2 sets with 30 and 40% 1RM of 8 and 6 repetitions, respectively, for the session with 50% 1RM; 2 sets with 40 and 50% 1RM of 6 and 4 repetitions, respectively, for the session with 60% 1RM; 3 sets with 40, 50, and 60% 1RM of 6, 4, and 3 repetitions, respectively, for the session with 70% 1RM; and 4 sets with 40, 50, 60, and 70% 1RM of 6, 4, 3, and 2 repetitions, respectively, for the session with 80% 1RM, respectively.

### Statistical Analysis

The data are presented as mean ± standard deviation (SD). The Shapiro-Wilk normality test was conducted to verify the distribution of each variable. Absolute reliability was assessed by test-retest and expressed in relative terms as an intrasubject CV, which was calculated as 100•SEM/mean, being SEM the standard error of measurement (root mean square of the intrasubject total mean square). Stokes (1985) stated that CV values below 15% can be labeled as “satisfactory”. A repeated-measures analysis of variances (ANOVA) was performed to analyze the differences between relative loads (50%, 60%, 70%, and 80% 1RM) for the different descriptive variables assessed through the set until failure. A 4 (%1RM) x 13 (VL thresholds) ANOVA was calculated for analysis of differences in the %Rep for each %1RM. When the interaction was significant, Bonferroni post hoc tests were utilized. The relationships between variables were analyzed by fitting a second-order polynomial to the given data, with coefficients of determination (R^2^) and standard error of estimate (SEE) being calculated. General %Rep-VL relationships were obtained by pooling together the data from all participants for each relative load, whereas an individual %Rep-VL relationship was determined specifically for each participant for every relative load. Statistical analyses were performed using SPSS for Mac (IBM Corporation, New York, NY) (release 20.0.0), with the level of significance set at 0.05.

## Results

### Evolution of Repetition Velocity throughout a Set Until Failure in the Bench-Press Exercise

The descriptive characteristics of each protocol are shown in Table 1. The fastest MPV values (MPV_BEST_) attained in each protocol matched the targeted MPV corresponding to each intensity. As expected, both MPV_BEST_ and the load lifted were significantly different among all loading conditions. MPV for all intensities was almost identical for the last repetition (MPV_LAST_). As the intensity increased, both the VL and the MNR significantly decreased.

**Table 1:** Descriptive characteristics for the bench-press sets performed to failure against different relative loads.

	50% 1RM (0.79 m•s^−1^)	60% 1RM (0.67 m•s^−1^)	70% 1RM (0.55 m•s^−1^)	80% 1RM (0.43 m•s^−1^)
Load (kg)	22.2 ± 4.1	25.4 ± 4.4^5^	29.5 ± 5.5^5,6^	33.6 ± 6.3^5,6,7^
MPV_BEST_ (m•s^−1^)	0.78 ± 0.02	0.67 ± 0.02^5^	0.54 ± 0.02^5,6^	0.43 ± 0.02^5,6,7^
MPV_LAST_ (m•s^−1^)	0.16 ± 0.05	0.16 ± 0.04	0.15 ± 0.06	0.16 ± 0.03
VL (%)	79.2 ± 5.8	76.5 ± 6.3	73.3 ± 9.9^5^	63.7 ± 9.1^5,6^
MNR (n)	43.3 ± 24.0	25.1 ± 7.7^5^	18.5 ± 5.2^5,6^	10.9 ± 4.0^5,6,7^

Data are mean ± standard deviation. 1RM: one-repetition maximum; Load: mass lifted in the corresponding session; MPV_BEST_: mean propulsive velocity of the fastest repetition in the set; MPV_LAST_: mean propulsive velocity of the last repetition in the set; VL: velocity loss incurred within the set; MNR: maximal number of repetitions completed in the set. Statistically significant differences with respect to: 50%1RM: ^5^ p ≤ 0.05; with respect to 60%1RM: ^6^ p ≤ 0.05; with respect to 70%1RM: ^7^ p ≤ 0.05

### Relationships between the Percentage of Performed Repetitions and the Percentage of Velocity Loss

**Figure 1:**
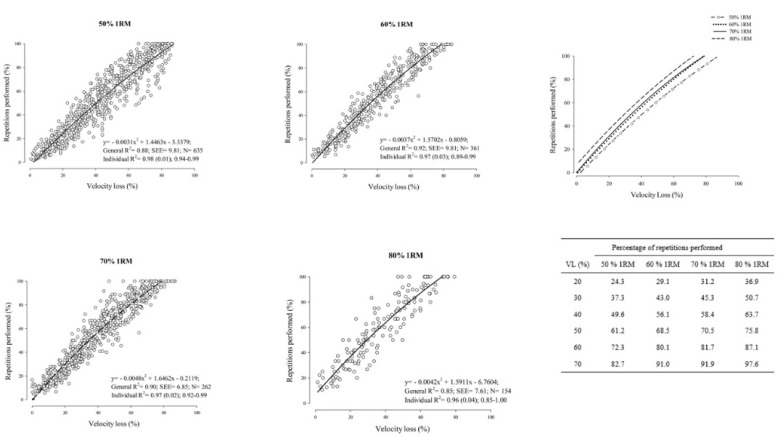
Relationships between the percentage of performed repetitions (%Rep) and the percentage of velocity loss (VL) for 50%, 60%, 70%, and 80% 1RM. The table on the right depicts the %Rep when a given magnitude of VL is reached for each intensity, obtained from the general equation.

Relationships between the %Rep and the percentage of VL for all the intensities analyzed are shown in Figure 1. High coefficients of determination (R^2^ = 0.85–0.92) and low SEE (6.85–9.81%) were observed for all the intensities analyzed. The mean individual R^2^ values were between 0.96 and 0.98 for all loading conditions (Figure 1).

Table 2 shows the description of the %Rep when a specific magnitude of VL was reached for each intensity. Thirteen magnitudes of VL were analyzed (from 10% to 70%). It was observed that for each intensity, as the percentage of VL increased, the %Rep progressively increased. Also, for a given magnitude of VL, the %Rep was higher as the intensity increased.

**Table 2:** Percentage of completed repetitions out of the maximum number of repetitions to failure when a given magnitude velocity loss is reached.

	Percentage of repetitions completed			
Velocity loss (%)	50% 1RM	60% 1RM	70% 1RM	80% 1RM
**10**	10.1 ± 5.3	14.6 ± 4.8	15.5 ± 6.1^5^	22.3 ± 7.8^5,6,7^
**15**	17.2 ± 6.0	22.1 ± 5.4^5^	22.9 ± 6.1^5^	30.1 ± 8.6^5,6,7^
**20**	24.2 ± 7.2	29.4 ± 6.1^5^	30.5 ± 6.4^5^	37.7 ± 9.9^5,6,7^
**25**	31.2 ± 8.5	36.6 ± 6.7^5^	37.6 ± 7.1	45.2 ± 11.2^5,6^
**30**	38.0 ± 9.5	43.6 ± 7.2^5^	44.7 ± 8.0	52.5 ± 12.2^5,6^
**35**	44.8 ± 10.4	50.5 ± 7.5^5^	51.8 ± 8.6	59.7 ± 12.8^5,6^
**40**	51.4 ± 11.0	57.2 ± 7.6^5^	58.8 ± 9.4	66.7 ± 12.9^5,6^
**45**	58.0 ± 11.3	63.7 ± 7.6^5^	65.6 ± 10.1	73.6 ± 12.8^5,6^
**50**	64.5 ± 11.4	70.2 ± 7.4	72.3 ± 10.9	80.3 ± 12.5^5,6,7^
**55**	70.9 ± 11.2	76.4 ± 7.1	78.9 ± 11.6	86.9 ± 12.2^5,6,7^
**60**	77.1 ± 10.8	82.5 ± 6.7	85.5 ± 12.4	93.3 ± 12.4^5,6,7^
**65**	83.3 ± 10.2	88.5 ± 6.3	91.8 ± 13.4	99.5 ± 13.4^5,6^
**70**	89.4 ± 9.6	94.3 ± 6.0	98.2 ± 14.5	

Data are mean ± SD. 1RM: one-repetition maximum. Statistically significant differences with respect to 50%1RM: ^5^ p ≤ 0.05; with respect to 60%1RM: ^6^ p ≤ 0.05; with respect to 70%1RM: ^7^ p ≤ 0.05

### Reliability of the Measurements

The MNR showed CV values of 16.1% and 20.8% for 50% and 80% 1RM, respectively (Table 3). Regarding the %Rep for a given VL, from 20% VL, CVs ranged from 6.3% to 14.6% being higher when the VL reached in the set was lower. However, 10% VL showed CV values of 34.3% and 20.8% for 50% and 80% 1RM, respectively.

**Table 3:** Absolute reproducibility (coefficient of variation: CV) for the percentage of completed repetitions with respect to the maximum number of repetitions (MNR) when a given percentage of velocity loss is reached with the loads of 50% and 80% 1RM.

	50% 1RM			80% 1RM			
Velocity loss (%)	Round 1	Round 2	CV (%)		Round 1	Round 2	CV (%)
**10**	10.1 ± 5.3	10.2 ± 4.6	34.3		22.3 ± 7.8	20.3 ± 11.8	20.8
**15**	17.2 ± 6.0	17.4 ± 5.7	19.6		30.1 ± 8.6	28.0 ± 12.4	15.7
**20**	24.2 ± 7.2	24.4 ± 7.2	14.6		37.7 ± 9.9	35.5 ± 13.0	13.9
**25**	31.2 ± 8.5	31.4 ± 8.6	12.3		45.2 ± 11.2	42.8 ± 13.5	13.1
**30**	38.0 ± 9.5	38.3 ± 9.8	10.6		52.5 ± 12.2	49.9 ± 13.8	12.4
**35**	44.8 ± 10.4	45.0 ± 10.8	9.8		59.7 ± 12.8	56.8 ± 13.8	11.6
**40**	51.4 ± 11.0	51.7 ± 11.6	8.9		66.7 ± 12.9	63.4 ± 13.6	10.6
**45**	58.0 ± 11.3	58.2 ± 12.1	8.0		73.6 ± 12.8	69.9 ± 13.2	9.7
**50**	64.5 ± 11.4	64.6 ± 12.6	7.2		80.3 ± 12.5	76.1 ± 12.4	8.8
**55**	70.9 ± 11.2	70.9 ± 13.1	6.6		86.9 ± 12.2	82.1 ± 11.4	8.2
**60**	77.1 ± 10.8	77.1 ± 13.6	6.2		93.3 ± 12.4	88.0 ± 10.2	8.1
**65**	83.3 ± 10.2	83.2 ± 14.5	6.1		99.5 ± 13.4	93.6 ± 9.0	7.5
**70**	89.4 ± 9.6	89.2 ± 15.7	6.3				
**75**	95.5 ± 9.2	95.0 ± 17.4	4.9				
**MNR**	43.3 ± 24.0	42.5 ± 20.0	16.1		10.9 ± 4.0	12.6 ± 5.3	20.8

Data are mean ± standard deviation. 1RM: one repetition maximum

## Discussion

The main findings of the study revealed a close relationship between the magnitude of VL in a set and the %Rep (R^2^ values of 0.85–0.92 and SEE ~6–10%). Likewise, better adjustments were obtained through individual %Rep-VL relationships (R^2^ values of 0.96–0.98). Furthermore, when a specific VL was reached, the %Rep revealed satisfactory absolute reliability from a certain VL threshold (>15% VL). Collectively, these findings highlight the utility of VL monitoring for determining the %Rep in the BP exercise in women. Nonetheless, it is important to note that VL higher than 15% is required to obtain acceptable reliability in this context.

Several studies conducted with male participants have consistently reported very close relationships between the magnitude of VL in a set and the %Rep in the BP exercise over a range of intensities from 50% to 85% 1RM (González-Badillo et al., 2017; Hernández-Belmonte et al., 2022; Rodríguez-Rosell et al., 2020; Sánchez-Moreno et al., 2021). The magnitudes of these relationships observed in men were higher (R^2^: 0.92–0.99) than the ones observed in our study in women (R^2^: 0.85–0.92) for the different common intensities examined (50%, 60%, 70%, and 80% 1RM). These differences could be explained in part by the higher strength level and BP background of male participants involved in the above-mentioned studies (relative strength normalized per kg of body mass for men: 1.07 ± 0.20 and 1.21 ± 0.18, in González-Badillo et al. (2017) and Sánchez-Moreno et al. (2021) compared to the female participants in our study, who exhibited relative strength of 0.69 ± 0.15). Even in the study of Hernández-Belmonte et al. (2022) who divided the whole group into three subgroups according to their relative strength ratio, the low relative strength ratio group in the BP was <1.10, which quite higher compared to our female group. According to previous research, women generally have lower values of absolute strength than men and such differences are more pronounced in the upper than in the lower limbs (Holloway and Baechle, 1990). Concerning relative strength, either related to body mass or to lean body mass, the differences between men and women in the squat exercise tend to disappear, while in the BP, these differences remain quite high (ratio = 0.59) (Bartolomei et al., 2021). Likewise, almost perfect individual %Rep-VL relationships (R^2^ values of 0.96–0.98) were observed. These findings align with data previously reported for men (Sánchez-Moreno et al., 2021). Sánchez-Moreno et al. (2021) observed in the BP exercise that %Rep-VL relationships adjusted individually showed higher R^2^ than general equations (0.97–0.99 vs. 0.80–0.94). Therefore, our findings indicate that employing VL magnitude can provide a precise approach to prescribing BP volume among women. However, individual relationships between VL magnitude and the %Rep could foster a better uniform degree of effort by individuals.

The MNR completed with each relative load (50–80 %1RM) was higher in our study conducted with women than that observed for men in the BP against the same relative loads (González-Badillo et al., 2017; Rodríguez-Rosell et al., 2020; Sánchez-Moreno et al., 2021). For instance, the MNR with 50% 1RM in the present study was 43.3 repetitions, while in studies conducted with men it was 25.2–28.1 repetitions (González-Badillo et al., 2017; Rodríguez-Rosell et al., 2020; Sánchez-Moreno et al., 2021). Although the relative intensities employed were not the same in the study of Hernández-Belmonte et al. (2022) (65, 75, 85, and 95% 1RM), our study with women reveals also a superior MNR. Previous research has found that the MNR correlates positively with the number of capillaries per mm^2^ of the muscle cross-sectional area (Terzis et al., 2008), but it correlates negatively with the percentage of type II fibers (Douris et al., 2006). Sex-based differences in skeletal muscle mass and fiber-type composition have been well documented (Janssen et al., 2000). Women frequently exhibit a more reduced muscle fiber cross-sectional area (Roberts et al., 2018) and a higher proportion of type I fibers relative to men (Miller et al., 1993). Hence, it appears that the differences in the MNR completed against a given load (%1RM) in women and men may depend, in part, on the specific muscle characteristics (Janssen et al., 2000; Roberts et al., 2018) and training background (Richens and Cleather, 2014). Another interesting point observed by Hernández-Belmonte et al. (2022) in all the exercises used, and specifically in the BP exercise, was that the MNR was higher at each specific VL as the strength level increased. This was accentuated when VL exceeded 30% and with the lowest intensities evaluated in the study (65% 1RM). This fact was observed when different strength levels were compared in a male population. In this regard, it is also remarkable that women performed a higher MNR than men against each relative load even if the MPV for each intensity was lower for women than the one used for men (i.e., 0.93 in men for 50% 1RM vs. 0.79 m•s^−1^ for women in our study) (González-Badillo et al., 2017; Rodríguez-Rosell et al., 2020; Sánchez-Moreno et al., 2021). That is, VL induced per repetition is lower in women compared to men.

Regarding the %Rep corresponding to a given VL, it has been stated that when individuals reach a 30% VL in a BP set against loads of 50–70% 1RM, they have completed ~50% of the MNR (González-Badillo et al., 2017; Rodríguez-Rosell et al., 2020; Sánchez-Moreno et al., 2021). However, in our study, it was observed that women needed to reach higher magnitudes of VL, approximately 40%, to complete half of the MNR in the BP for the same range of intensities. This suggests that women present higher endurance to VL than men, since for a given VL women are further from task failure. The potential physiological mechanisms underlying higher endurance for women have been described in the previous paragraph. Therefore, VL thresholds that have shown effectiveness in improving strength performance in men may not be equally effective for women. This assumption arises from the fact that when men and women perform the BP with a similar VL magnitude, they experience a different level of effort. Therefore, specific sex equations are required for prescribing RT volume from VL monitoring.

Interestingly, regarding the %Rep for a specific magnitude of VL, they were quite similar for 60% and 70% 1RM intensities, slightly lower for 50% 1RM, and slightly higher for 80% 1RM (Table 2 and Figure 1). These findings agree with previous research conducted with men reporting that the relationship between the magnitude of VL in the set and the %Rep depends on the relative load being lifted (González-Badillo et al., 2017; Rodríguez-Rosell et al., 2020). Therefore, the development of VL throughout the set is slightly different in the distinct relative loads. Thus, equations for each relative load (50–80% 1RM) were provided to estimate the level of effort achieved by women during a BP set (Figure 1).

Regarding reliability, our results reveal that VL higher than 15% is required to obtain “satisfactory” reliability when prescribing BP volume by the VL approach in women. These reliability values are lower than those previously observed by González-Badillo et al. (2017), who reported greater absolute reliability (within-subject CV ≤ 6.6%) between two sessions separated by 6–7 days, regarding the %Rep achieved at different VL (from 15% to 75%) during BP exercise performed on a Smith machine with a 60% 1RM load in men. However, Sánchez-Moreno et al. (2021) found reliability values (within-subject CV ≤ 17.7%) similar to those observed in the present study for the %Rep achieved at different VL magnitudes (from 20% to 85%) in the BP exercise on a Smith machine in resistance-trained men. Those authors reported within-subject CV values ranging from 13.0% to 21.4% for 15% VL (Sánchez-Moreno et al., 2021). These values are similar to those observed in our study (CV values for 15% VL: 15.7–19.6%).

The lack of absolute reliability observed for low VL thresholds could be explained by the technical demands inherent in this method. At times, it may be observed that between the first and the second repetition, there does not exist a significant difference in the MPV attained, especially when using light loads. A possible explanation for this could be that fatigue is still low and coexists with potentiation (Blazevich and Babault, 2019), which may result in some variability in VL development. Consequently, in the initial phases of the set, significant differences in the percentages of experienced VL (e.g., 0% vs. 10% VL) at the initial %Rep values (i.e., 5-10-15% Rep) can be observed, leading to an increase in CV values. However, as the set progresses, fatigue develops resulting in higher MPV drops. This situation is reflected in the evolution of the CV during the set, which decreases as the %Rep approaches the maximum possible (Gonzalez-Badillo et al., 2017; Sánchez-Moreno et al., 2021). However, recent observations suggest that setting low and moderate VL thresholds (e.g., 10%, 20%, and 30% VL) aids in managing fatigue responses to RT and shows reproducibility (Weakley et al., 2024).

## Conclusions

This study provides novel insight into the velocity-based RT approach for monitoring and prescribing the resistance exercise stimulus for women. In conclusion, the main finding of this study was the high relationship between the magnitude of VL within a set and the %Rep for all the intensities examined in the BP exercise performed by women (R^2^ values of 0.85–0.92), which underscores the effectiveness of using VL as a tool for prescribing BP volume in women. Furthermore, individual %Rep-VL relationships provided even better adjustments (R^2^ values of 0.96–0.98), highlighting the need for personalized training approaches. These relationships differ from those previously observed in men for the BP exercise, which confirms that sex-specific differences in %Rep-VL relationships would allow coaches to tailor training guidelines according to sex characteristics since a given VL threshold means a different level of effort in men and women. Likewise, the %Rep completion when a specific magnitude of VL is reached in a set revealed only satisfactory absolute reliability from a certain VL threshold (>15% VL). These results reinforce the magnitude of the VL attained in a set as a tool for monitoring BP volume in women, especially when VL is prescribed via individual %Rep-VL relationships and higher VL magnitudes than 15% are attained.
